# Morphology Control in Waterborne Polyurethane Dispersion Nanocomposites through Tailored Structure, Formulation, and Processing

**DOI:** 10.1021/acs.langmuir.5c00226

**Published:** 2025-04-18

**Authors:** Garrett M. Abrahamsen, Zoe A. B. Lequeux, Lisa K. Kemp, Dane N. Wedgeworth, James W. Rawlins, John K. Newman, Sarah E. Morgan

**Affiliations:** † School of Polymer Science and Engineering, University of Southern Mississippi, 118 College Drive # 5050, Hattiesburg, Mississippi39406, United States; ‡ US Army Corps of Engineers (USACE), Engineer Research and Development Center (ERDC), Vicksburg, Mississippi39180, United States

## Abstract

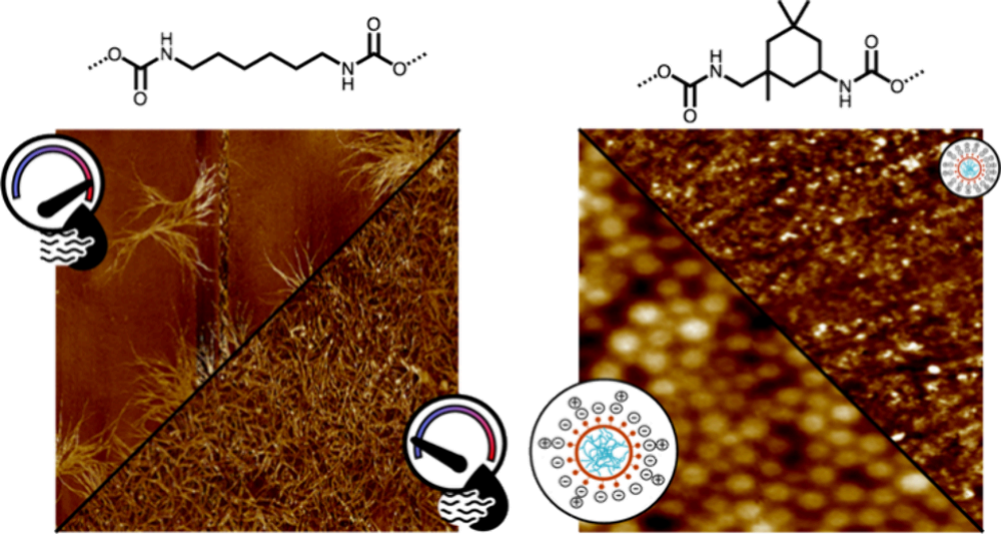

Waterborne polyurethane dispersions (PUDs) have garnered increasing interest in recent years due to the growing demand for environmentally friendly materials. The unique phase-separated morphologies exhibited in PUD films and coatings provide opportunities for directing the distribution of functional additives and controlling properties. Although there has been extensive research on polyurethanes for several decades, the mechanisms underlying the PUD morphology formation are poorly understood. The morphologies are driven by interactions between hard segments (HS), and the process is further complicated by the presence of colloidal particles and the intricate interaction between the urethane/urea linkages and water. In this work, structure–property-processing relationships between HS content and structure, relative humidity, particle size, and the resulting dry film morphology of PUDs were determined in two diisocyanate systems: hexamethylene diisocyanate (HDI), a symmetric, flexible diisocyanate; and isophorone diisocyanate (IPDI), an asymmetric, sterically hindered cyclic diisocyanate. HDI-based films exhibited semicrystalline morphologies with HS superstructures that are sensitive to relative humidity. IPDI-based films displayed spherical coalescence-suppressed morphologies influenced by particle size and zeta potential. PUD compositions and processing conditions were controlled to produce nanocomposite films with an enhanced dispersion of nanoadditives.

## Introduction

Polyurethanes (PUs) are widely used across most major industries, including medicine, automotive, aerospace, and consumer products.^1,2^ Their application versatility stems from the tunability of their mechanical properties, chemical resistance, and thermal conductivity, achieved by varying the structures of the diisocyanates and polyols used in their synthesis. Waterborne polyurethane dispersions (PUDs) are a type of PU that is dispersed in water, typically through the incorporation of an internal emulsifier.^3−5^ Besides providing the environmental benefit of reducing volatile organic content (VOC) in their formulation, they also possess high flexibility, excellent water resistance, good film formation properties, and tailorable morphology. One method of PUD synthesis involves the use of acetone as a cosolvent to help control the viscosity during prepolymer formation, which aids in chain extension and neutralization.^3,6^ This method has also been shown to reduce the reactivity between NCO and NH groups, while ensuring excellent reproducibility and easy removal after dispersion.^7,8^ The physical properties of PUs can be modified based on the degree of microphase separation between polyol soft segments (SSs) and diisocyanate (DI) hard segments (HSs), which occurs due to their thermodynamic incompatibility.^9^ Researchers focused on controlling microphase separation have been successful through multiple methods, including using selective solvents based on solubility parameters,^10^ adjusting HS content,^11,12^ and varying DI chemical structure and symmetry.^13,14^

Further organization of this microphase separation on larger scales, which can lead to the formation of superstructures,^15,16^ is heavily dictated by the degree of hydrogen bonding between HSs.^14^ Morphologies achievable through the association of HSs are globules (free, unorganized HS) and spherulitic structures (organized HS) on the micron-scale, and fibrillar (nonchain folded HS) and lamellar (chain folded HS) structures on the nanoscale.^17^ Aneja et al. investigated spherulitic superstructure morphologies of PU, where the HS lengths were controlled to be 1–4 repeat units.^15^ The study revealed that the spherulitic structures were not comprised of chain-folded lamella-shaped hard domains but were hard ribbon-like domains, and their connectivity increased with higher HS length and HS content. Bonab et al. increased the HS content of p-phenylene diisocyanate-based PU and surpassed the percolation threshold of HS domains, leading to increased hydrogen bonding.^11^ These changes were linked to the presence of a greater number of physical cross-links between HS structures, and increased interconnections of the HS were observed by atomic force microscopy (AFM). Although hydrogen bonding was found to contribute to the formation of these structures, no relationship between thermal transitions and film morphology was identified, as the resulting organized structures lacked melting endotherms. The presence of water can further complicate the already complex morphology-forming mechanisms. A study by Serkis et al. of drying procedures reported that slow water evaporation followed by heating at 50 °C led to the higher organization of rod-like HS structures, which indicated that the organization of HSs depends not only on the rate of water evaporation but also on the drying temperature.^18^

The process of drying films cast from colloidal suspensions is also a crucial step in determining the film morphology. During this process, the colloidal particles undergo several physical changes, including particle coalescence, that can ultimately influence the properties and morphology of the final film. The glass transition temperature (*T*_g_) of the colloidal particles is a significant factor influencing particle coalescence during film drying.^19^ The minimum temperature required for film formation is dependent on the *T*_g_ of the polymer, where full coalescence of colloidal particles is less likely to occur at drying temperatures below the *T*_g_ of the polymer. Acrylic suspensions have been reported that did not coalesce, presenting honeycomb-like morphologies of packed particles with defined grain boundaries.^20^ Although hindered coalescence can have an adverse effect on films, such as creating friable discontinuous films,^21^ it has also been shown that this organized packing can be used to localize conductive additives to the interface and reduce the additive concentration necessary to achieve a percolated pathway.^22,23^ This honeycomb morphology has been observed in acrylate/methacrylate latexes synthesized from emulsion polymerization,^24−26^ but to our knowledge, it has not been reported for PUDs.

The incorporation of carbonaceous nanomaterials such as carbon nanotubes or graphene into polymer matrices has gained attention for the potential to enhance physical, thermal, and conductive properties. Nanoscale dispersion of the additive is crucial, as aggregated particles are known to reduce properties such as ductility and toughness.^27,28^ The organization of the nanoadditive into a percolated network has been shown to improve thermal and electrical conductivity.^29−32^ Gradient composites, which feature varying filler concentrations, have been used to tailor properties like conductivity, especially in electromagnetic interference (EMI) shielding applications.^33−35^

In this study, we report the effects of DI structure, HS content, particle neutralization, triethylamine (TEA): 2,2 bis(hydroxymethyl)propionic acid (DMPA) mole ratios, and relative humidity (RH) during drying on the morphology of PUD films and the resulting effect on nanoadditive dispersion. Poly(tetramethylene ether)glycol (PTMEG) was chosen as the polyol for polymerization with two DIs, hexamethylene diisocyanate (HDI) and isophorone diisocyanate (IPDI). HDI has a symmetric molecular structure, an even number of connected atoms along the backbone, and flexible aliphatic linear bonds, all of which aid in the generation of crystalline hard phases and phase separation from soft domains.^36^ IPDI, on the other hand, has an asymmetric structure, pendant primary −CH_3_ substituent groups, and a reduced solubility parameter (closer to that of PTMEG) compared to HDI, all of which contribute to reduced phase separation and a higher *T*_g_.^37^ Hydrogen bonding properties, thermal properties, and morphologies of HDI and IPDI-based PUD films were analyzed and demonstrated clearly distinguishable differences resulting from the phase separation behavior and propensity to form crystalline structures. Nanocomposite films were prepared with Fe_3_O_4_-reduced graphene oxide nanoadditives, and their morphologies were determined by optical and scanning electron microscopy (SEM). This study defines new processing methods to produce waterborne polyurethane coatings with controlled morphologies and the distribution of electromagnetic nanoadditives for advanced applications.

## Experimental Section

### Materials

HDI, IPDI, TEA, dibutyltin dilaurate (DBTDL), ethylenediamine (EDA), ammonium hydroxide (28–30%), cetyltrimethylammonium bromide (CTAB), iron(III) chloride hexahydrate (FeCl_3_·6H_2_O), hexadeuterodimethyl sulfoxide (DMSO-d6), iron(II) sulfate heptahydrate (FeSO4·7H_2_O), and hydrazine monohydrate 64% (N_2_H_4_·H_2_O) were all purchased from Sigma-Aldrich and used without further modification. PTMEG (*M*_n_ = 2000 g/mol, OH number 1000, Sigma-Aldrich) and DMPA (Sigma-Aldrich) were dried in a vacuum oven under reduced pressure for 12 h at 90 and 60 °C, respectively. 1,4-Butanediol (BDO) (Sigma-Aldrich) and acetone (Sigma-Aldrich) were dried over 4 Å sieves for 3 days before use to remove residual water. Single-layer graphene oxide nanoplatelets (flake size 0.5–5 μm) were purchased from Graphene Supermarket.

### Instrumentation

#### Fourier Transform Infrared Spectroscopy (FTIR)

Urethane polymerization was tracked using a ReactIR 4000 (Mettler Toledo). The area of a two-point baseline absorption peak centered at 2274 cm^–1^ (associated with isocyanate (NCO) stretching) was used to calculate the % of unreacted diisocyanates.^38^ When the change in peak area had plateaued and reached the expected % of unreacted diisocyanates for the prepolymer, the chain extender was added, and unreacted diisocyanates were tracked until the % diisocyanate was at or below 16.7%. The ReactIR probe was then removed, and the polyurethane solution was neutralized.

FTIR spectra of dried PUD films were obtained using a Thermo Scientific Nicolet iS50 FT-IR instrument in ATR mode. Reported spectra are the average of 128 scans at a resolution of 2 cm^–1^ and were normalized to the PTMEG ether peak (1108 cm^–1^).^39^

#### Nuclear Magnetic Resonance (NMR) Spectroscopy

NMR spectra were obtained at 25 °C in DMSO-d6 with a 600 MHz Bruker Avance III (TopSpin 3.1p17) spectrometer by taking an average of 64 scans (delay of 5 s). Chemical shifts for ^1^H NMR spectra were referenced to signals from DMSO-d6 (δ = 2.50 ppm) within the MestReNova software.

#### X-ray Photoelectron Spectroscopy (XPS)

XPS was used to analyze the structural characteristics of the graphene-based additive. Spectra were obtained from a Thermo-Fisher ESCALAB Xi+ spectrometer equipped with a monochromatic Al X-ray source (1486.6 eV). The additive was finely spread on double-sided copper tape and adhered to the sample holder. Measurements were performed using the standard magnetic 61-lens mode and charge compensation. The base pressure in the analysis chamber during spectral acquisition was 3 × 10^–7^ mBar. Spectra were collected at a takeoff angle of 90° from the plane of the surface. The pass energy of the analyzer was set at 20 eV for high-resolution spectra and 150 eV for survey scans, with energy resolutions of 0.1 and 1.0 eV, respectively. Binding energies were calibrated with respect to C 1s at 285.3 eV. All spectra were recorded using Thermo Scientific Avantage software; data files were translated to VGD format and processed using the Thermo Avantage package v5.9904.

#### Thermogravimetric Analysis (TGA)

Thermal stability experiments were conducted utilizing a TA Instruments Discovery 550 TGA. The additive was placed within high-temperature platinum pans and heated under nitrogen (flow rate of 20 = mL/min). The temperatures were increased at a heating rate of 10–1000 °C.

#### Differential Scanning Calorimetry (DSC)

Modulated DSC (MDSC) experiments were conducted by equilibrating the sample at −90 °C and heating the sample to 200 °C at a heating rate of 3 °C/min, with an amplitude of ±1.0 °C and a period of 90 s. Samples were either directly cooled to −90 °C or treated to initial heating to 200 °C before being cooled for the MDSC experiment. MDSC thermograms are split into three separate signals^40^: one signal from linear changes in temperature (total heat flow), and two signals from sinusoidal heating rate corresponding to transitions from changes in heat capacity (reversing heat flow) and transitions that are independent of changes in heating rate (nonreversing). All data were recorded and processed in TA Trios software.

#### Dynamic Mechanical Analysis (DMA)

A DMA 850 (TA Instruments, Inc.) was used with the setting of strain control in tension mode, and free films cut from the samples (0.8 mm thick, 5.3 mm wide) were analyzed at a frequency of 1.0 Hz with a heating rate of 3.0 °C/min. Storage modulus (*E*′) and loss tangent (tan δ) were analyzed from −100 to 100 °C for all samples. The *T*_g_ of the sample was defined as the tan δ maxima.^41^ All data were recorded and processed in TA Trios software.

#### Dynamic Light Scattering (DLS)

Particle size and zeta potential were obtained using a Malvern Instruments Zetasizer Nano ZS (633 nm incident wavelength, 173° scattering angle) operating at 25 °C. Samples were diluted with DI water to concentrations of 0.5–1 mg/mL. If significant aggregation was apparent for the dispersions, samples were sonicated for 10 min and remeasured.

#### Atomic Force Microscopy (AFM)

To obtain PUD film morphology, AFM images of cast PUD films were collected using a Bruker Dimension Icon 3000 scanning probe microscope in tapping mode within a temperature (23 °C) and humidity (50%) controlled room using a standard Veeco RTESP silicon probe (cantilever length, 125 μm; nominal force constant, 40 N/m; resonance frequency, 350 kHz). Height and phase images were collected simultaneously. Scans were done one month after casting to ensure the achievement of equilibrium film morphology. Images were processed by using Nanoscope Analysis software.

#### Scanning Electron Microscopy (SEM)

Analysis of the morphology of cryo-fractured surfaces of films was done by utilizing a Helios G4 UC field emission scanning electron microscope with an 8.5 mm working distance (FE-SEM, Thermo-Fisher Scientific, Waltham, MA). The elemental maps and the distribution of FRGO across the corresponding SEM images were characterized through the attached X-Max energy dispersive X-ray spectroscopy system (EDS, Oxford Instruments, Abingdon, United Kingdom). The working distance of the microscope is dictated by the geometry of the detector mounted in the SEM chamber.

#### ImageJ Analysis of SEM Images

ImageJ was used to analyze the SEM data following literature procedures.^42,43^ The normalized average gray values, which indicate the brightness of a pixel, were taken from the EDS Fe map images, where darker colors, such as the red Fe signals from the EDS overlays, increased the gray values. These values were plotted from the bottom to the top of composite films and were fitted using either a fully linear line function or a line function composed of both an exponential and a linear portion, named “Exponential Linear” within the OriginPro software (Figure S8). Coefficients and slopes obtained from the line equations are listed in Table S7. A detailed procedure with example images can be found within the Supporting Information (Figure S9).

### Synthesis

#### Synthesis and Phase Inversion of Waterborne PUDs

PUDs were synthesized using the acetone method.^3,6^ Six different PUDs were synthesized with varying hard segment structures, using either HDI or IPDI as the DI; and hard segment weight % (HS%) was varied between 30, 40, and 50%.

NCO/OH/NH_2_ mole ratios were kept constant at 1.2/1/0.12, and DMPA content was kept constant at 5 wt % of solid content. TEA:DMPA mol ratio was set to 1.1:1 for all PUDs, except for two PUDs containing IPDI with 50 HS%, where the TEA:DMPA mol ratio was 0.75:1 and 0.25:1. For a representative reaction, IPDI with 30 HS% (IPDI 30) was synthesized by first adding 20 g of PTMEG and 1.43 g of DMPA to a four-necked flask equipped with a condenser, mechanical stirrer, a ReactIR probe, and rubber septa. N_2_ gas was passed through a desiccant column and into the reaction vessel. The vessel was heated in an external oil bath to 80 °C, and the mixture was stirred at 400 rpm. Once a homogeneous mixture was formed, the temperature was lowered to 75 °C, and acetone was added to dissolve the reagents. IPDI (6.60 g) was then added dropwise using a needle syringe through the septa. Afterward, five drops of DBTDL were added. After 30.4% of unreacted NCO was reached, as determined by the area of the NCO stretching peak measured by ReactIR, the prepolymer was further chain extended with the addition of 0.37 g of BDO. The chain extension was also monitored by ReactIR, and the temperature was lowered to 50 °C once the reaction reached 16.7% NCO. Next, the equivalent amount of TEA (1.1:1 TEA:DMPA mol ratio) was added, and the neutralization was continued with stirring for 30 min. The temperature was then lowered to 40 °C, and a water/EDA solution (0.18 g of EDA in 183.4 mL of DI water) was added dropwise with 700 rpm stirring. The reaction mixture increased in viscosity until enough water was added to reach phase inversion, at which point the observed viscosity dropped significantly. The reaction was then mixed for another 30 min. After the additional mixing time, acetone was removed from the mixture by rotary vacuum distillation at 40 °C and below 300 mPa. The final solid wt % of the dispersion was adjusted to 25%.

#### Fe_3_O_4_-Reduced Graphene Oxide Saturated with Surfactant (FRGO) Synthesis

FRGO magnetic additive was prepared by coprecipitation of Fe^3+^ and Fe^2+^ ions within an aqueous suspension of GO based on a literature procedure.^44^ The molar ratio of Fe^3+^/Fe^2+^ was kept at 2:1. The GO suspension (175 mL at 5 mg/mL) was mixed with 2.04 g of FeCl_3_·6H_2_O and heated to 80 °C within a round-bottomed flask equipped with a water-cooled condenser and septa. The mixture was sparged with N_2_ gas for 15 min with mechanical mixing. FeSO_4_·7H_2_O (1.05 g) was then added, and the reaction vessel was sealed. NH_4_OH solution (14.8 N) was added dropwise with constant mixing until the pH of the reaction was ≥10 tracked with a Fisherbrand Accumet AB150 pH benchtop meter, and the reaction visually darkened. This solution was stirred at 80 °C for 4 h, after which 5.5 g of N_2_H_4_·H_2_O solution was added for chemical reduction of GO. The reduction mixture was allowed to stir for 24 h at 80 °C under a nitrogen atmosphere. This mixture was then washed six to seven times with deionized water and ethanol, centrifuged at 4600 rpm for 10 min, and the pelleted solid was collected. The supernatant was then filtered through a nylon membrane (0.20 μm pore size) under vacuum, and residual solids were isolated and washed several times with ethanol and DI water. The collected solid was combined and dried in a vacuum oven at 80 °C for 12 h. The dried solid was added to DI water at a concentration of 2 mg/mL, and CTAB was added under mixing. The CTAB was set at a 20:1 weight ratio to solid. This solution was ultrasonicated for 60 min at 60 W. The centrifuge and filtration procedures were repeated with only water washes to obtain a solid saturated with CTAB surfactant. FRGO was then freeze-dried for 48 h, and the powder was stored within a scintillation vial until further use.

### PUD Film Preparation

#### Neat PU Film Preparation

Films were prepared for morphological analysis by pouring the dispersions into Teflon drying dishes and drying at room temperature in atmospheric conditions, or within controlled environments with RH values of either <25 or >75% RH. After 10–14 days, these films were placed under vacuum for 24 h to remove any residual water. Films were further analyzed with AFM, DSC, and FTIR. The final thicknesses of the dried films were determined with the use of calipers and found to range from 0.8 to 1.2 mm. Films were kept in a desiccator between tests.

#### Composite PU FRGO Film Preparation

2 wt % FRGO composites were made by ultrasonically mixing 82.5 mg of FRGO and 10 mL of DI water until homogeneous (approximately 60 min). The FRGO dispersion was added to 15 mL of PUD and mixed with a homogenizer at 6000 rpm for 15 min. The new PUD-FRGO mixture was used to prepare films by pouring the dispersions into Teflon drying dishes. These were placed in a desiccator under nitrogen flow to maintain a constant humidity of <25% RH and to promote drying of the films at room temperature. After 10–14 days, these films were placed under reduced pressure for 24 h to remove any residual water. The final thicknesses of the dried films were determined with the use of calipers and found to range from 0.8 to 1.2 mm. Films were kept within a desiccator between tests. Samples for SEM characterization were submerged in liquid nitrogen for 5 min, then cryofractured by snapping the sample in half with two tweezers.

## Results and Discussion

### Structural Characterization

**Scheme 1:**
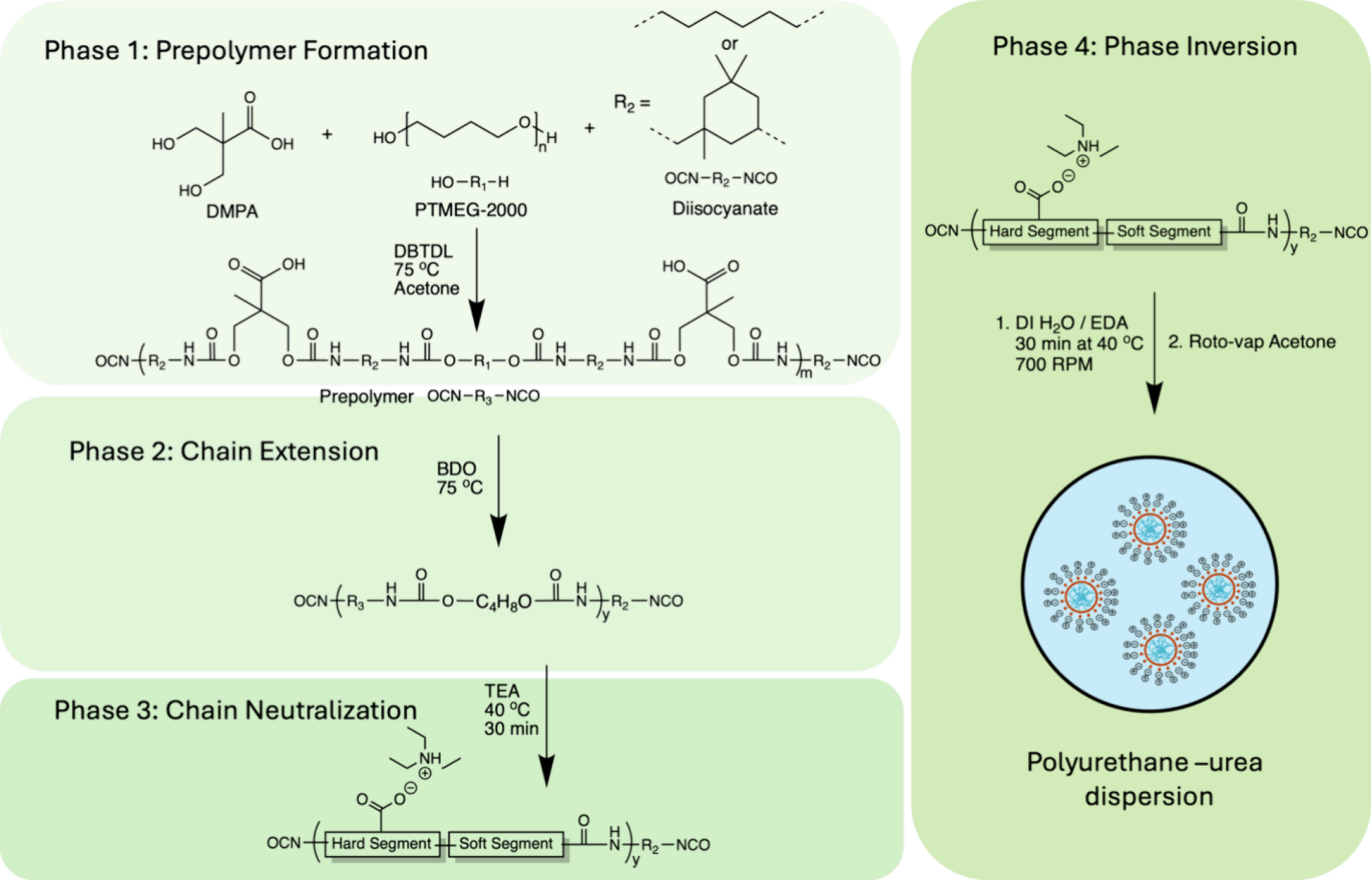
PUD Synthesis Including Steps of Prepolymer Formation, Chain Extension, Neutralization, and Phase Inversion

PUDs utilizing HDI as the DI were synthesized with HS content of 30, 40, and 50 as shown in Scheme 1, and dried at three different relative humidities of <25, ∼55, and >75%. The HDI PU samples are named by the DI monomer, HS content, and relative humidity during drying. For example, the sample synthesized using 30 wt % HDI at a relative humidity of ∼55% is HDI 30_55RH_.

PUDs utilizing IPDI as the DI were synthesized in a similar fashion to the HDI samples with the same HS content (wt %) of 30, 40, and 50. Mole ratios of TEA:DMPA were adjusted to create different particle sizes: (a) 1.1:1, (b) 0.75:1, and (c) 0.25:1. The IPDI PU samples are named by the DI monomer, HS content, and the ratio of TEA to DMPA. For example, the sample synthesized using 30 wt % IPDI at a 1.1 ratio of TEA:DMPA is IPDI 30_1.1TEA_.

The measured particle size and zeta potential of the PUDs are shown in Table S1. All PUDs with a 1.1:1 TEA:DMPA display a consistent particle size below 60 nm and have zeta potentials ≤−30 mV, which indicates the formation of stable dispersions.^45,46^ The average particle size was increased from 38 nm for IPDI 50_1.1TEA_ to 130 nm for IPDI 50_0.75TEA_ and 360 nm for IPDI 50_0.25TEA_. Typical ^1^H NMR spectra for HDI and IPDI samples are shown in Figure S1, and mole ratios for the products are shown in Table S2, showing good agreement between target and actual composition (Figure S1 and Table S2).

**Figure 1:**
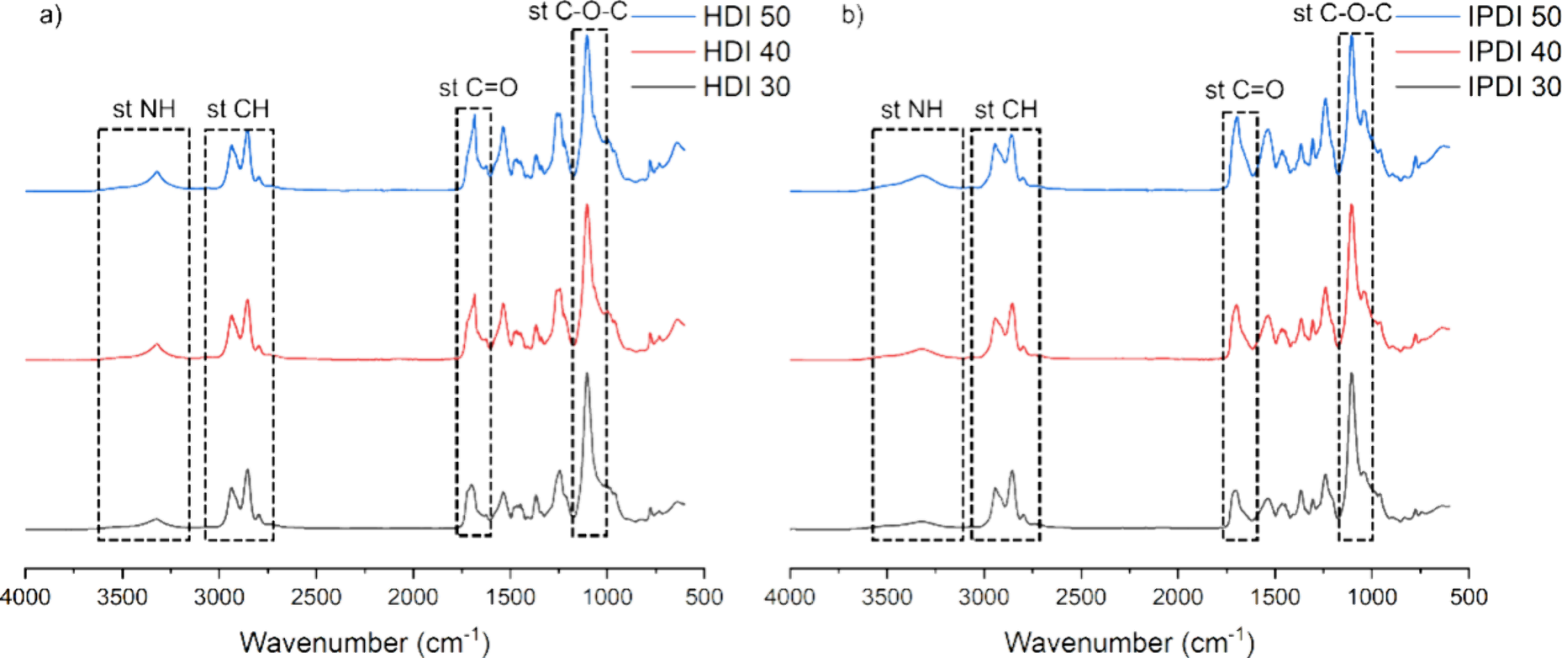
Overlayed ATR-FTIR spectra of cast PUD films containing either (a) HDI or (b) IPDI hard segments at HS contents of 30, 40, or 50%.

FTIR analysis was performed to better understand the chemical structure within the cast PUD films (Figure 1). Both HDI and IPDI spectra display peaks representative of the functional groups found within polyurethane dispersions (−NH stretching, C=O stretching, −O– stretching). The lack of an −NCO stretching peak near 2270 cm^–1^ in all samples indicates that full conversion was achieved and there are no detected residual −NCO groups. HDI samples show a sharp −NH peak at ∼3300 cm^–1^, attributed to hydrogen bonding between urea/urethane linkages within HS domains, and indicating a high degree of phase separation between HS and SS domains.^47^ IPDI samples, in contrast, show broad −NH peaks around 3300 cm^–1^, indicating lower levels of HS hydrogen bonding and greater HS/SS phase mixing. Side reactions between the carboxylic acid on DMPA and the isocyanate groups are possible. However, the reactivity between the carboxylic acid and the isocyanate group is much lower compared with the reactivity between the primary hydroxyl groups on the DMPA and the isocyanates. Steric hindrance of the DMPA also mitigates the reaction with the carboxylic group.^48,49^ Furthermore, the acetone method requires less internal emulsifier to form stable dispersions, thereby reducing the likelihood of side reactions.^8^ The particle size, NMR, and FTIR data suggest that any side reactions were minimal and did not negatively affect the dispersibility or stability of the PUDs.

### Thermal Properties

**Figure 2:**
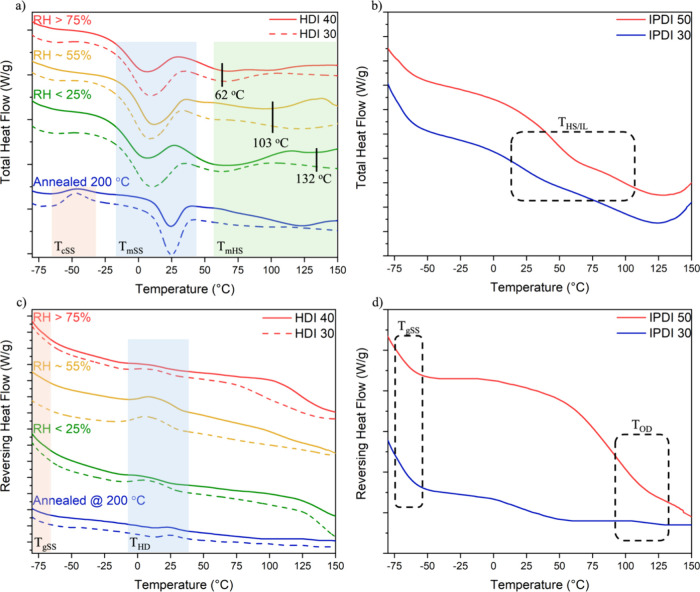
MDSC thermograms. HDI samples dried under different humidity conditions or after annealing at 200 °C: (a) Total heat flow and (c) reversible heat flow. IPDI produced under standard conditions: (b) total heat flow and (d) reversible heat flow.

In complex semicrystalline systems, second-order transitions can be difficult to identify with conventional DSC thermograms.^50^ MDSC analysis was conducted to more precisely analyze both crystalline and second-order thermal transitions in our systems. HDI and IPDI total heat flow and reversing heat flow thermograms are compared in Figure 2, with transitions for the HDI samples shown in Table S3. HDI films show that SS melting (*T*_mSS_) is not greatly affected by RH or HS wt % with *T*_mSS_ values of approximately 15 °C for all HDI films (Figure 2a). In contrast, the HS melting (*T*_mHS_) is split into three endothermic peaks and is shown to be influenced by both RH and HS wt %. These endotherms correspond to distinct HS crystal populations.^51^ In general, HS melting endotherms at higher temperatures are associated with more organized structures in PU.^51,52^ In our system, greater HS ordering is observed for systems dried under low humidity and with a higher HS content. This can be seen in the shift of *T*_mHS_ from about 60 °C in samples dried in 75% RH to around 130 °C in samples dried in 25% RH (Figure 2a). The higher *T*_mHS_ observed for HDI 40 is attributed to the formation of more organized domains and increased long-range order within the HS crystal lattice at higher HS content.^53−55^ In addition, when the HDI film (∼55% RH) is annealed at 200 °C, the SS and HS both become more organized, resulting in an increase in the *T*_mSS_ and *T*_mHS_. There is also the appearance of the SS crystallization (*T*_cSS_) transition in the range −50 °C, which can only be observed in annealed samples (Figure 2a).

The HDI *T*_gSS_ is observed between −60 and −70 °C in reversing heat thermograms (Figure 2c), and an additional HDI exothermic peak, related to hard domain ordering (*T*_HD_), is apparent. Kong et al. reported an exotherm below *T*_mHS_ for methylene diphenyl diisocyanate (MDI) TPU that they attributed to the rearrangement of HS into nuclei and metastable structures, which indicates the development of phase-separated HS domains.^56^ In our system, MDSC analysis suggests that the *T*_HD_ exotherm is related to HS nucleus formation. On annealing, T_HD_ shifts to a higher temperature. It has been proposed that the annealing of PU films removes most crystalline structures, but the organized HS nuclei remain.^56^ The nuclei facilitate the growth of the crystalline phase, where recrystallization is unimpeded by RH in the annealing process.

IPDI films containing a 1.1:1 TEA:DMPA ratio show no SS melting peaks in the total heat flow (Figure 2b), but a new transition is observed (shown more clearly in Figure S2). The origin of this transition, observed only in IPDI systems, is a matter of debate, and it is unclear if it represents the glass transition of amorphous HS or the dissolution of an interlayer (IL) between SS and HS domains (*T*_HS/IL_).^57^ Increasing the HS content in IPDI samples causes this transition to shift to higher temperatures, while the breadth of the transition is attributed to varied HS lengths. The temperature range of the T_HS/IL_ transition in our system is similar to that reported for *T*_g_s of IPDI TPU at 30–50 HS content.^58^ Similar to the HDI system, IPDI *T*_gSS_ is observed between −60 and −70 °C in the reversing heat thermograms (Figure 2d), and an additional transition is observed for IPDI 50. This transition has been attributed to an order/disorder transition (*T*_OD_) for hydrogen-bonded HS, and its breadth is representative of large distributions of HS lengths with varying degrees of organization.^55^ It has been demonstrated in latexes that films dried above their *T*_g_ produce full particle coalescence, while latexes dried below their *T*_g_ produce hindered coalescence.^23^ IPDI 50 was chosen to explore the effect of the TEA:DMPA ratio on particle coalescence, as this sample demonstrated more prominent thermal transitions in the MDSC analysis. In summary, a lowered RH of drying, annealing, and increased HS content all result in more highly organized HS structures with higher crystalline melting temperatures.

### Dynamic Mechanical Properties

**Figure 3:**
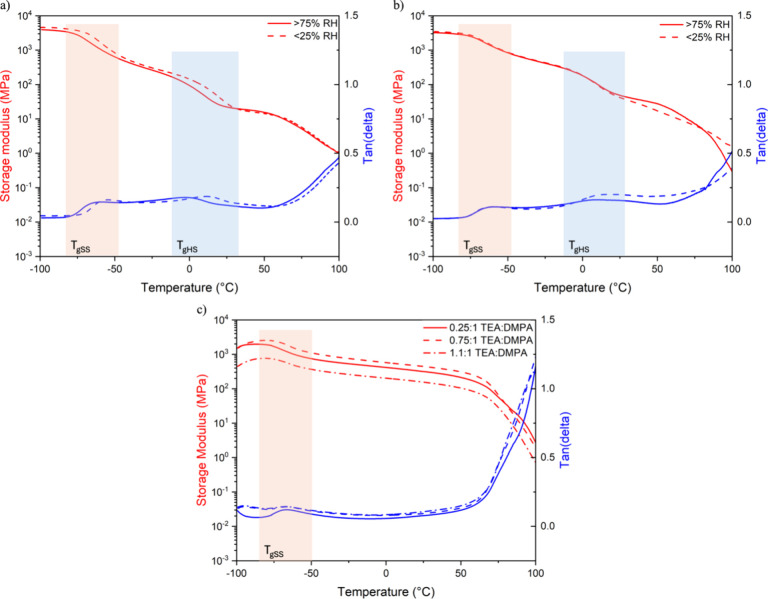
DMA thermograms. (a) HDI 30 dried under different humidity conditions, (b) HDI 40 dried under different humidity conditions, and (c) IPDI 50 produced with varying TEA:DMPA ratios.

DMA was conducted to determine the moduli and *T*_g_s of the materials. Plots of *E*′ and tan δ as a function of temperature are shown in Figure 3. Figure 3a,b represents HDI 30 and HDI 40 films dried under different relative humidities, and Figure 3c shows IPDI 50 prepared with different TEA:DMPA ratios. *T*_g_s and onset temperatures of the flow regime are shown in Table S4. The HDI films are flexible in comparison to the IPDI films, which are stiff and brittle. This observation is consistent with the modulus of the HDI and IPDI samples at room temperature (Figure 3), where the order of magnitude higher moduli of the IPDI samples indicate their higher stiffness. Tan δ peaks for the HDI 30 and HDI 40 films dried at <25 and >75% RH reveal two separate thermal transitions below room temperature, corresponding to the *T*_g_s of the SS and HS phases. SS *T*_g_, in the range of −57 ±3 °C, is relatively unchanged by HS content or drying condition, but HS *T*_g_, in the range of −3 to 20 °C, is affected by both. At a given drying condition, *T*_gHS_, is 10 °C greater for HDI 40 than for HDI 30. For both HDI 30 and HDI 40 samples, *T*_gHS_ is ∼10 °C higher when dried at <25% RH than at >75% RH. Onset flow temperatures are also slightly higher for samples dried at <25%. These findings are consistent with the MDSC data (Figure 2a), where it was observed that both *T*_mHS_ and *T*_gHS_ increase as the crystalline domain size and order increase.

As expected, DMA of the IPDI 50 samples with varying TEA:DMPA ratios shows only one distinct thermal transition related to the SS. IPDI SS *T*_g_, in the range of −66 ±1 °C, is relatively unchanged by TEA:DMPA ratio. The SS *T*_g_ of the HDI samples is 10 °C greater than the SS *T*_g_ of the IPDI samples. This is attributed to the influence of the SS crystalline domains in the HDI samples, increasing the *T*_g_ in comparison to the *T*_g_ observed for the fully amorphous SS in the IPDI films.^59^ A decrease in the TEA:DMPA ratio leads to a stiffer and more brittle film, as shown in Figure 3c, where the modulus of the sample with the highest TEA:DMPA ratio (1:1:0) is lower than that of the other samples. This observation is in agreement with reports that an increase in ductility is caused by the interdiffusion of particles and the formation of entanglements of the polymer chains in fully coalesced latex samples.^60^

### AFM Analysis

**Figure 4:**
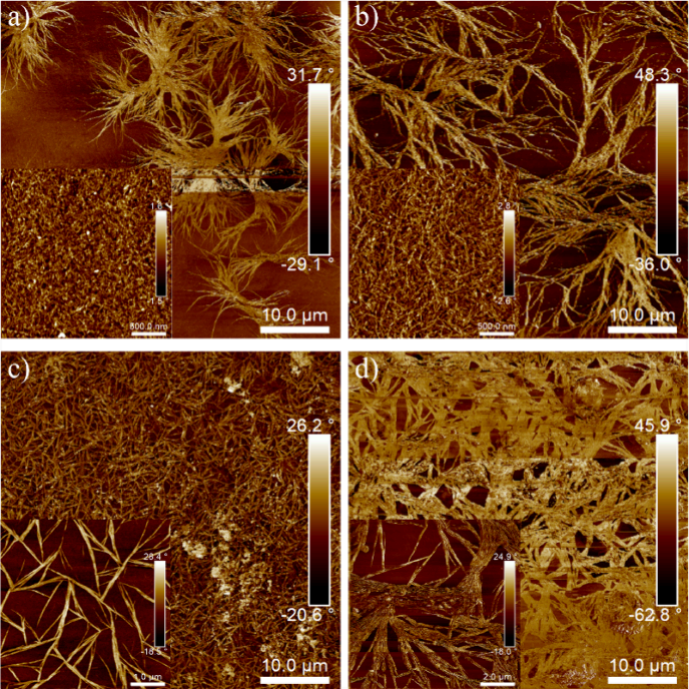
AFM phase images (50 μm × 50 μm) of (a) HDI 30_75RH_, (b) HDI 40_75RH_, (c) HDI 30_25RH_, and (d) HDI 40_25RH_. The insets are expanded views of crystalline regions (a, b) at 5 × 5 μm and (b, c) at 10 × 10 μm.

AFM images of HDI 30 and HDI 40 films dried at <25 and >75% RH are shown in Figure 4. The HDI 50 sample did not form uniform films; therefore, it was not possible to obtain images of that sample. The HDI 30 and HDI 40 samples display bright fibrillar features that are attributed to crystalline structures. As AFM scans were collected at 23 °C, above the SS melting peak observed in MDSC thermograms (Figure 2c), we attribute these features to HS crystallites. Crystallinity is higher in the HDI 40 than in the HDI 30 sample, and films prepared under low humidity conditions (<25% RH) display larger and more continuous crystalline features than those prepared at high humidity. Low-humidity samples show needle-like crystals, with a higher degree of fibril packing in HDI 40_25RH_. High-humidity samples, on the other hand, display sheaf-like structures attributed to the initial stages of spherulite growth.^61^ The structures are believed to be composed of hydrogen-bonded HSs that develop into nuclei that support the growth of the sheaf-like structures.^56^

**Figure 5:**
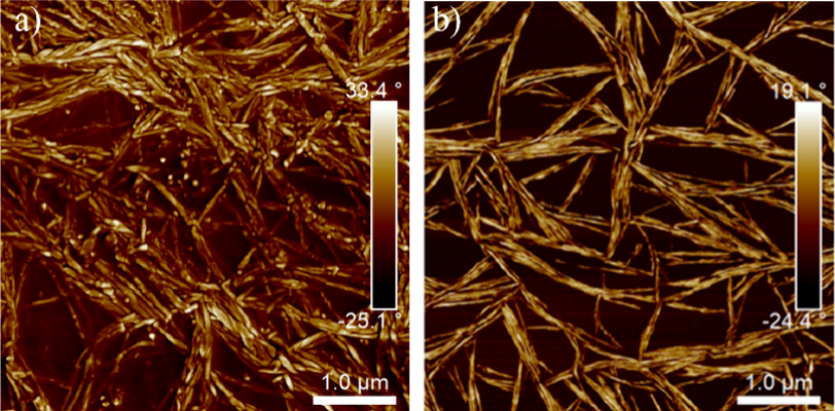
AFM phase images (5 × 5 μm) of (a) HDI 30_75RH_ and (b) HDI 30_25RH_ after annealing at 60 °C for 3 h.

Figure 5 shows AFM images of HDI 30_75RH_ and HDI 30_25RH_ films annealed at 60 °C for 3 h, near the HS melting point observed in MDSC thermograms (Figure 2c). Both samples show an increase in rigid fibrils with greater levels of interconnection and fibril packing than those observed in the unannealed samples. Morphologies observed in AFM are consistent with the crystalline behavior determined by DSC.

**Figure 6:**
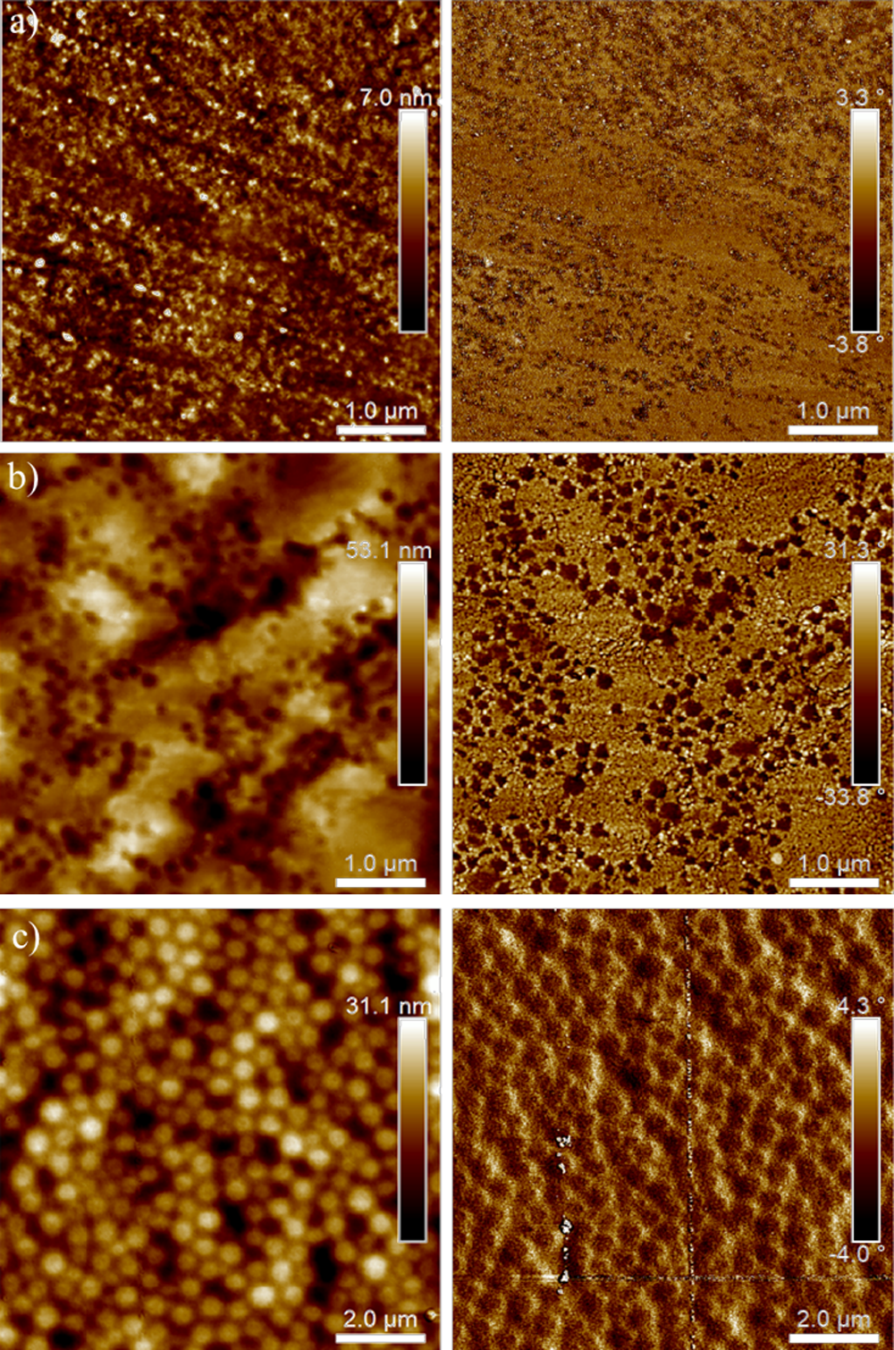
AFM height (left) and phase (right) images of (a) IPDI 50_1.1TEA_ (5 × 5 μm), (b) IPDI 50_0.75TEA_ (5 × 5 μm), and (c) IPDI 50_0.25TEA_ (10 × 10 μm).

AFM height and phase images of IPDI 50 samples prepared with varying molar ratios of TEA:DMPA are shown in Figure 6. IPDI 50_1.1TEA_ appears amorphous with no clear phase-separated features (Figure 6a). A similar amorphous morphology is observed for IPDI 30_1.1TEA_, as shown in Figure S3. IPDI 40_1.1TEA_ showed properties identical to those of IPDI 30_1.1TEA_, so it was not imaged. IPDI 50_0.75TEA_ displays a mixed morphology with bright regions of uncoalesced particles and dark coalesced regions (Figures 6b and S4). IPDI 50_0.25TEA_ shows a packed particle honeycomb-like morphology (Figure 6c). This sample also showed the largest particle size in the DLS evaluation. The lack of particle coalescence is partially attributed to the larger particle sizes and more negative zeta potentials observed for the samples prepared with reduced TEA levels (Table S1), which could reduce coalescence due to particle–particle repulsion. The high-temperature thermal transition (62 °C) observed for IPDI 50 in MDSC analysis, which hinders chain motion at the drying temperature, may also inhibit particle coalescence (Figure 2b).

### Graphene-Based Nanoadditive Distribution

FRGO was synthesized as described in the experimental section. XPS of FRGO (Figure S5a) shows distinct peaks that are associated with the Fe 2p_3/2_ and Fe 2p_1/2_ states found in Fe_3_O_4_ nanoparticles. Atomic concentration (%) (Table S5) shows the appearance of Fe and Br absent in neat GO, which indicates successful decoration of the GO with Fe_3_O_4_ and CTAB surfactant. The area % of the C 1s deconvoluted peaks (Figure S5b and Table S6) shows an increase in the area % of the C–C peak, which is expected due to the presence of CTAB. SEM/EDS images (Figure S6) of FRGO also confirm the presence of Fe on the surface. TGA (Figure S7) of the FRGO shows initial weight loss steps caused by moisture evaporation below 120 °C, removal of residual oxygen groups and CTAB surfactant adhered to the surface of GO between 120 and 240 °C, and a broad step from 240 to 475 °C from the decomposition of the carbon skeleton of GO.^62−64^

**Figure 7:**
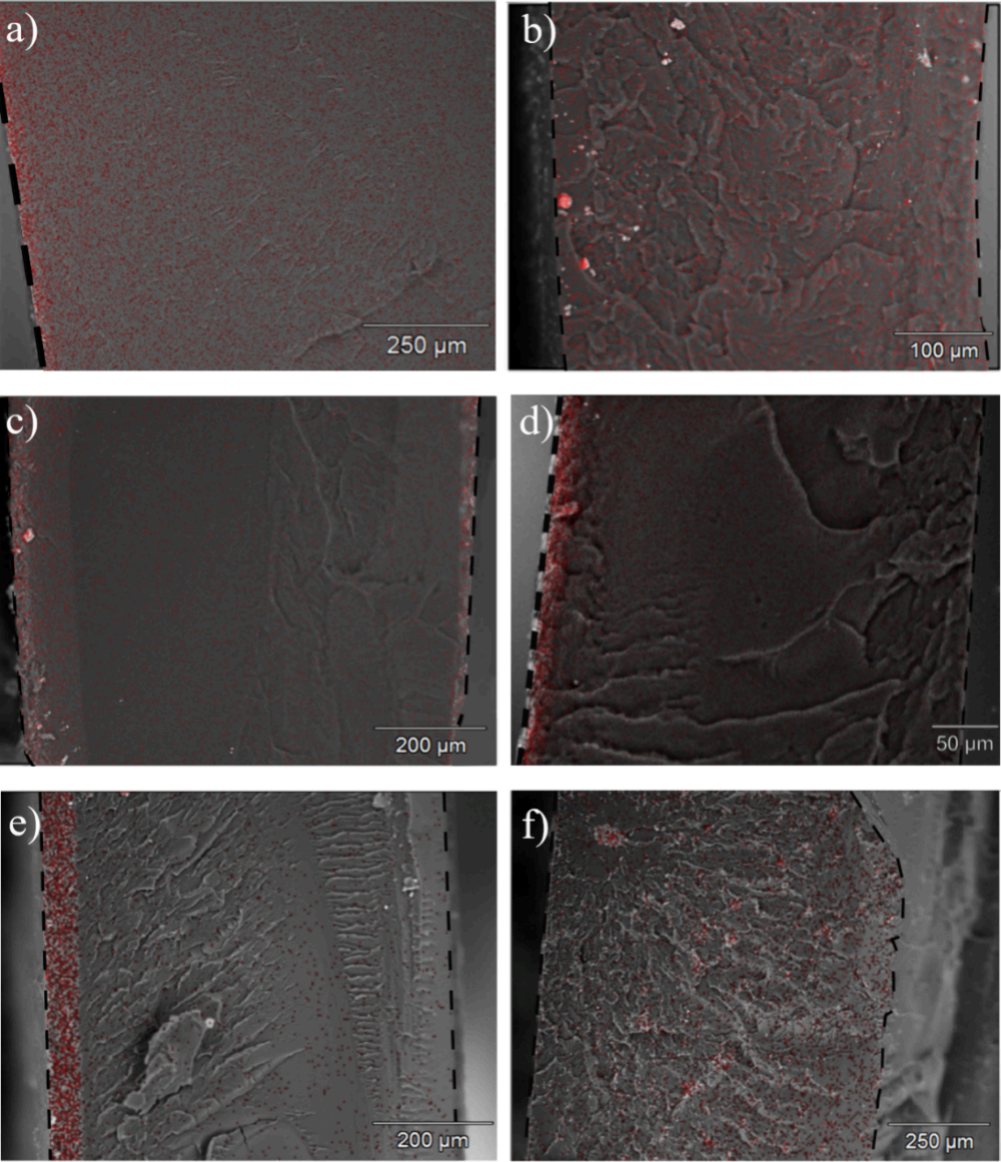
SEM images of cryo-fractured cross sections with EDS Fe overlay for (a) HDI 30_25RH_, (b) HDI 40_25RH_, (c) IPDI 30_1.1TEA_, (d) IPDI 50_1.1TEA_, (e) IPDI 50_0.75TEA_, and (f) IPDI 50_0.25TEA_. Films are oriented with the bottom of the films on the left side of the images and the top of the films on the right-hand side.

Films containing 2 wt % percent FRGO were prepared in selected PUD matrices as described in the experimental section. SEM/EDS images of HDI composite films are shown in Figure 7a,b. HDI 30_25RH_ (Figure 7a) shows a higher concentration of Fe at the bottom of the sample (left-hand side of the image), indicating sedimentation of the FRGO during drying, while HDI 40_25RH_ shows a homogeneous distribution of the additive. This difference is attributed to the higher level of crystallinity and greater ordering of crystalline structures in HDI 40_25RH_ as compared to HDI 30_25RH_, which likely directs the ordering of FRGO to the interface of the crystallites and reduces sedimentation.

The SEM/EDS images of IPDI composites are shown in Figure 7c–e. IPDI 50_0.25TEA_ shows a homogeneous distribution of FRGO. In contrast, IPDI 50_1.1TEA_ and IPDI 50_0.75TEA_ show significantly higher Fe concentration at the bottom of the films, indicating sedimentation. IPDI50_0.25TEA_ is the only PUD film that displays hindered coalescence morphology through the entirety of the film surface, as shown in AFM images of neat PUD films (Figure 6c). It is hypothesized that the honeycomb-like morphology creates a volume exclusion effect that prevents the settling of FRGO to the bottom of the film, because the filler becomes trapped between the grain boundaries of the PUD particles. ImageJ was used to provide a quantitative assessment of FRGO distribution,^42,43^ as described in the experimental section. Analysis and fitting of the data reveal that HDI 40_25RH_ and IPDI 50_0.25TEA_ have a linear distribution of nanoparticles across the film, while the other samples show an exponential drop in nanoparticle concentration with depth from the film substrate (Figures S8, S9, and Table S7). These findings support the conclusions drawn based on AFM analysis. The zeta potential differences between PUDs and FRGO (Table S1) may also contribute to increased electrostatic interaction with the more positively charged FRGO particles, leading to decreased sedimentation.^65−67^

## Conclusions

We have identified processing and synthetic strategies that produce reproducible morphologies in waterborne PUD films that enhance the dispersion of nanoadditives. For the linear HDI system, increased HSC results in larger HS domains and a higher degree of crystalline network formation, with crystallinity behavior controlled by the RH during drying. The most highly organized crystalline structures yield the greatest FRGO dispersion. For the asymmetric cyclic isocyanate system, the morphology of the films depends on the particle size, which is dictated by the TEA:DMPA mole ratio but is independent of drying RH. The film containing the highest IPDI concentration with the lowest TEA:DMPA mole ratio yielded a previously unreported hindered coalescence morphology that also yielded the greatest dispersion of FRGO particles. These findings enhance our understanding of PUD morphology formation mechanisms and provide design guidelines for creating nanocomposite coatings with controlled additive localization in an environmentally friendly waterborne medium.

## Supporting Information Available

Table S1: particle size and zeta potential of synthesized PUDs and FRGO from DLS. Figure S1: ^1^H NMR spectra; Table S2: Comparison of theoretical and experimental PTMEG/HDI mol ratios obtained from feed molar ratio and ^1^H NMR; Table S3*:* MDSC thermal transitions and enthalpy values; Figure S2*:* Total heat flow thermograms; Table S4: DMA mechanical transitions; Figure S3: AFM images of IPDI 30_1.1TEA_ samples; Figure S4: AFM images of IPDI 50_0.75TEA_ samples; Figure S5: XPS spectra of Fe 2p peak and deconvoluted C 1s peaks of FRGO additives; Table S5: Area % of C 1s deconvoluted peaks for FRGO; Table S6: Atomic % of N, C, O, Fe, and Br for FRGO; Figure S6: SEM images of with EDS overlay of FRGO; Figure S7: Thermal decomposition curves of FRGO obtained from TGA; Figure S8: Normalized average gray values from EDS images vs distance from the bottom of films; Table S7: Line functions used to fit gray values including the different rates of gray value changes and percentages of change in total gray value (GV) due to each part of the line (exponential and linear portions); and Figure S9: Example of steps of data analysis process utilizing ImageJ (PDF)
