# Supplementary material for: Morphology Control in Waterborne Polyurethane Dispersion Nanocomposites through Tailored Structure, Formulation, and Processing
Source: Langmuir. 2025 Apr 18;41(16):10383–93. doi: 10.1021/acs.langmuir.5c00226 (PMC12044684; doi:10.1021/acs.langmuir.5c00226)
Supplement: Supplementary file 1 — la5c00226_si_001.pdf [file la5c00226_si_001.pdf]

Supporting Information

Morphology control in waterborne polyurethane dispersions nanocomposites through tailored structure, formulation, and processing

Garrett M. Abrahamsen[1 ¥], Zoe A.B. Lequeux[1 ¥], Lisa K. Kemp[1], Dane N. Wedgeworth[2], James W. Rawlins[1], John K. Newman[2], Sarah E. Morgan[1]*

[1]School of Polymer Science and Engineering, University of Southern Mississippi, 118 College Drive # 5050, Hattiesburg, MS 39406, USA

[2] Engineer Research and Development Center (ERDC), US Army Corps of Engineers (USACE), Vicksburg, MS 39180, USA

[¥] These authors contributed equally

*Corresponding author: sarah.morgan@usm.edu

Table S1. Particle size and zeta potential of synthesized PUDs and FRGO from DLS

Sample*	Avg. Particle Size (nm)	PDI	Avg. Zeta Potential (mV)
HDI 30_{55RH}	41±1	0.25	-42±0.7
HDI 40_{55RH}	57±0.5	0.39	-44±2.5
HDI 50_{55RH}	58±1	0.46	-41±2.1
IPDI $30_{1.1TEA}$	39±0.3	0.06	-57±1.4
IPDI $40_{1.1TEA}$	49±0.7	0.08	-52±2.6
IPDI $50_{1.1TEA}$	38±0.5	0.11	-48±3.3
IPDI $50_{0.75TEA}$	129 (Bi-Modal: 69.8 & 290 nm) ±0.9	0.30	-65±1.5
IPDI $50_{0.25TEA}$	359±4.05	0.24	-72±1.0
FRGO	-	-	-10±4.5

*DLS measurements were done in triplicate for each dispersion. The average and standard deviation (+/-) are reported.

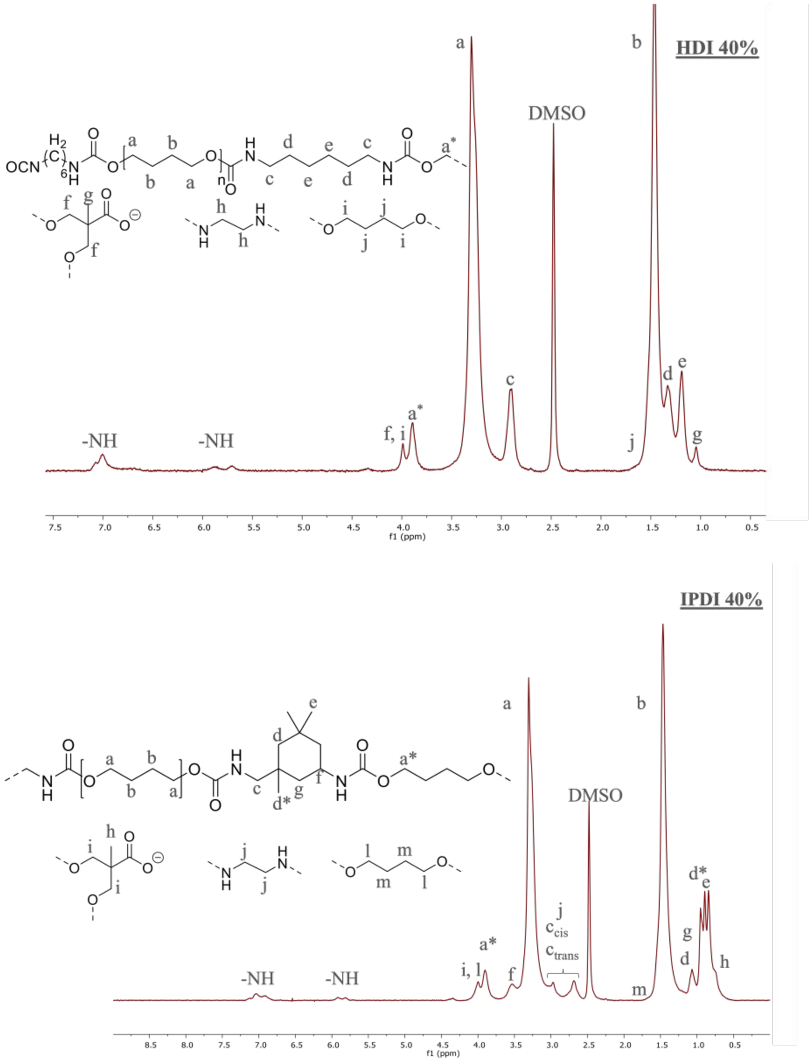

Figure S1. ^{1}H NMR spectra of (a) HDI 40_{55RH} and (b) IPDI $40_{1.1TEA}$

Table S2. Comparison of theoretical and experimental PTMEG/HDI mol ratios obtained from feed molar ratio and ^{1}H NMR, respectively.

Sample	Theoretical PTMEG/HS	Experimental PTMEG/HS*
HDI 30_{55RH}	0.28	0.25
HDI 40_{55RH}	0.18	0.20
HDI 50_{55RH}	0.12	0.12
IPDI $30_{1.1TEA}$	0.34	0.33
IPDI $40_{1.1TEA}$	0.22	0.19
IPDI $50_{1.1TEA}$	0.15	0.16

*PTMEG/HS mol ratios were obtained from peaks at 2.8 and 1.4 ppm for HDI and PTMEG respectively, and the IPDI cis and trans peaks at 3.0 and 2.6 ppm.

Polyurethane composition was confirmed by calculating the experimental hard/soft block molar ratio through NMR peak integration. The mols of PTMEG and HS were determined by normalizing the peak labeled ‘c’ for both the HDI and IPDI in Figure S1 to the theoretical number of protons present based on how many moles of HS were added to the reaction. The PTMEG peak labeled ‘b’ was then integrated to calculate the mols of PTMEG in the polymer.

Table S3. MDSC thermal transitions and enthalpy values

Sample	RH	T_{mSS} (°C)	ΔH_{mSS} (J/g)	T_{mHS} 1 (°C)	ΔH_{mHS} 1 (J/g)	T_{mHS} 2 (°C)	ΔH_{mHS} 2 (J/g)	T_{mHS} 3 (°C)	ΔH_{mHS} 3 (J/g)
HDI 30	>75%	10.2	22.0	61.5	8.6	-	-	-	-
HDI 30	~55%	10.9	26.2	49.0	2.8	114.5	5.4	-	-
HDI 30	<25%	10.9	22.5	60.0	8.6	-	-	141.0	0.3
HDI 40	>75%	6.1	15.4	62.4	4.3	99.5	1.7	129.0	0.2
HDI 40	~55%	14.5	22.1	39.0	10.2	102.9	1.4	-	-
HDI 40	<25%	6.1	16.2	64.0	25.6	-	-	132.0	1.6

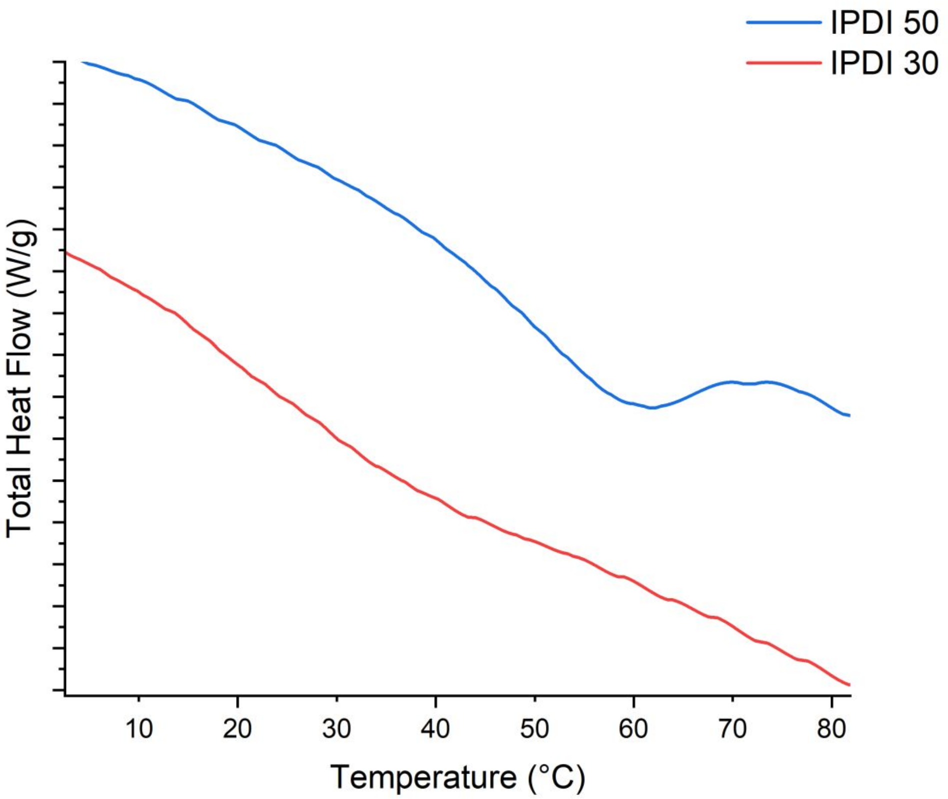

Figure S2. MDSC total heat flow thermograms of IPDI $30_{1.1TEA}$ and IPDI $50_{1.1TEA}$ between 0 and 80°C.

Table S4. DMA mechanical transitions

**Sample**	**RH**	**T_{gSS} (°C)**	**T_{gHS} (°C)**	**Flow Onset (°C)**
HDI 30	>75%	-58.6	-2.6	66.1
HDI 30	<25%	-54.4	10.9	70.4
HDI 40	>75%	-57.9	9.5	82.5
HDI 40	<25%	-60.1	19.7	84.3
**Sample**	**TEA:DMPA Ratio**	**T_{gSS} (°C)**	**T_{gHS} (°C)**	**Flow Onset (°C)**
IPDI 50	1.1:1	-67.0	-	65.2
IPDI 50	0.75:1	-65.6	-	70.7
IPDI 50	0.25:1	-66.1	-	82.2

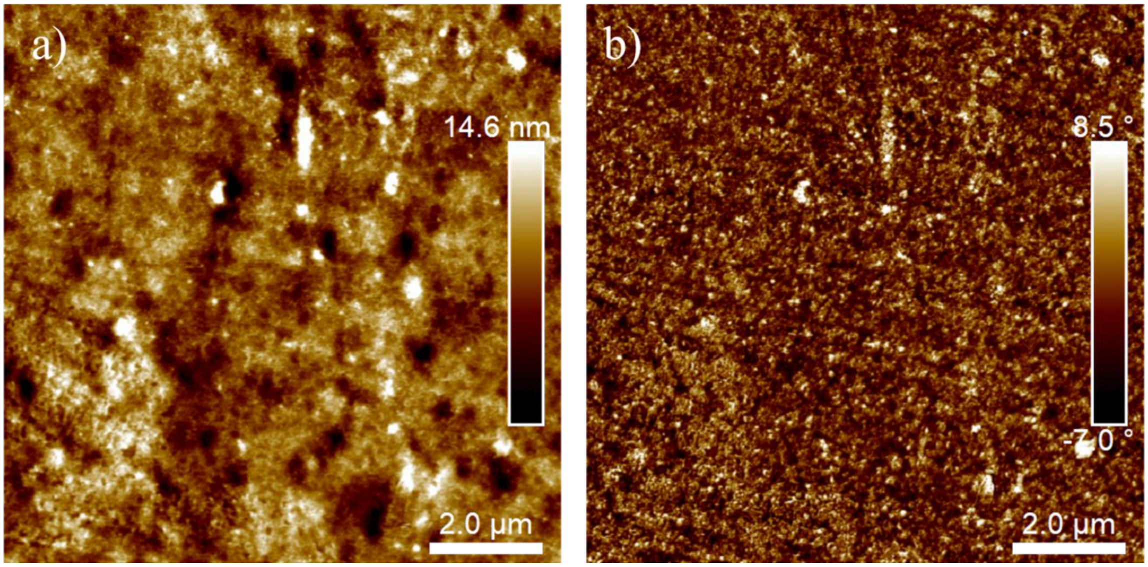

Figure S3. AFM (a) height and (b) phase images of IPDI $30_{1.1TEA}$ samples (10 x 10 µm)

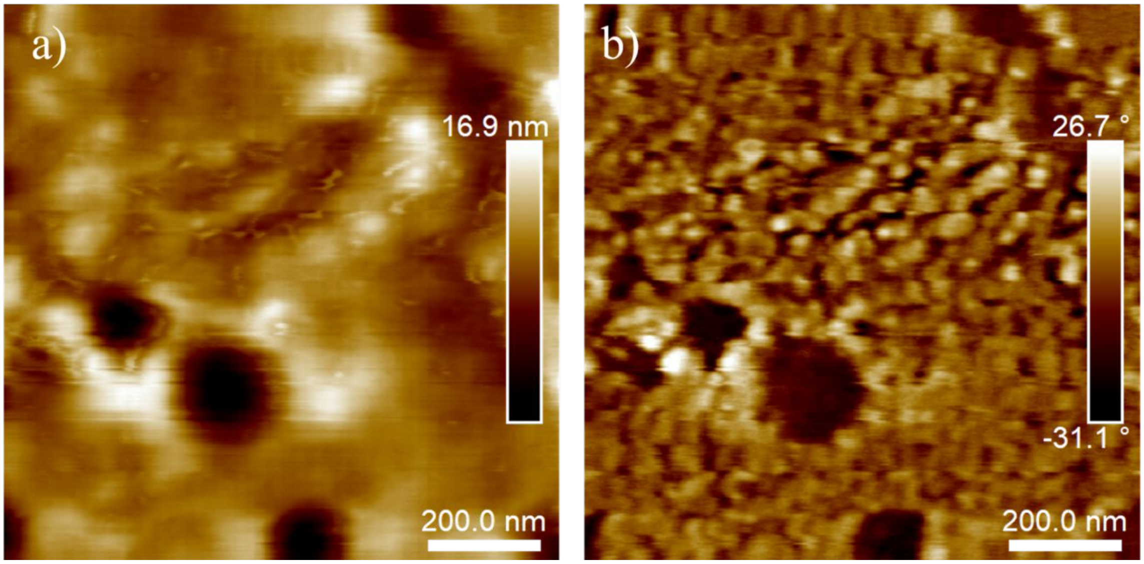

Figure S4. AFM (a) height and (b) phase images of IPDI $50_{0.75TEA}$ (1 x 1 µm)

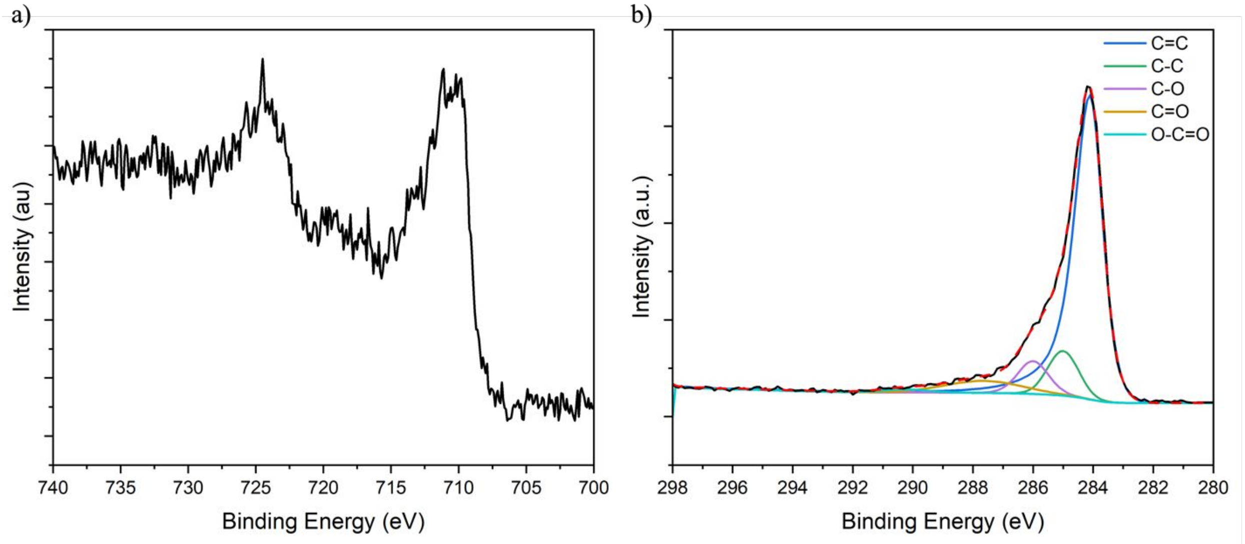

Figure S5. XPS spectra of (a) Fe2p peak and (b) deconvoluted C1s peaks of FRGO additive.

Table S5. Atomic % of N, C, O, Fe, and Br

Addtive	N%	C%	O%	Fe%	Br%
Graphene Oxide	0.8%	64.0%	35.0%	-	-
FRGO	4.0%	80.0%	11.0%	1.6%	1.0%

Table S6. Area % of C1s deconvoluted peaks

Peak	C=C	C-C	C-O	C=O	O-C=O
**Area %**	55%	20%	15%	7.3%	2.6%

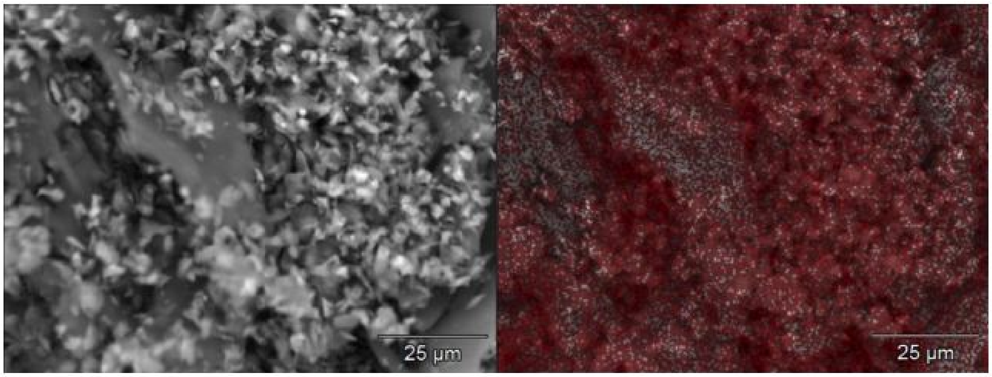

Figure S6. SEM images of a) FRGO with b) EDS overlay displaying Fe concentration as red dots.

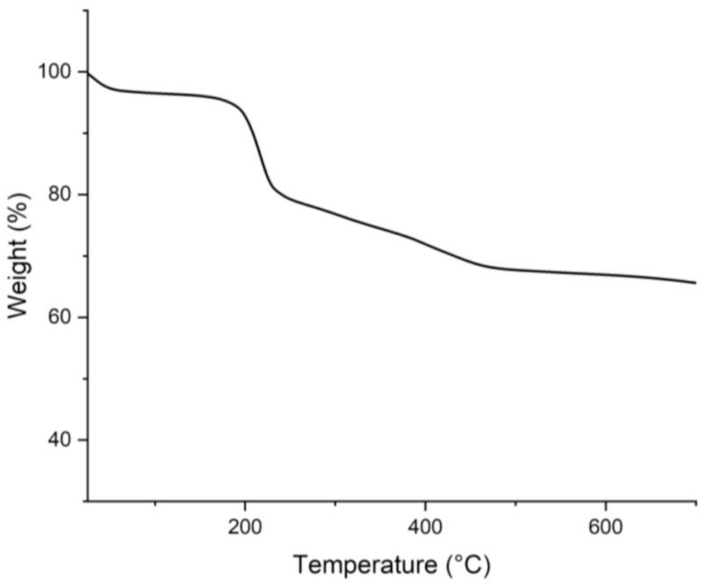

Figure S7. TGA thermal decomposition curves of FRGO

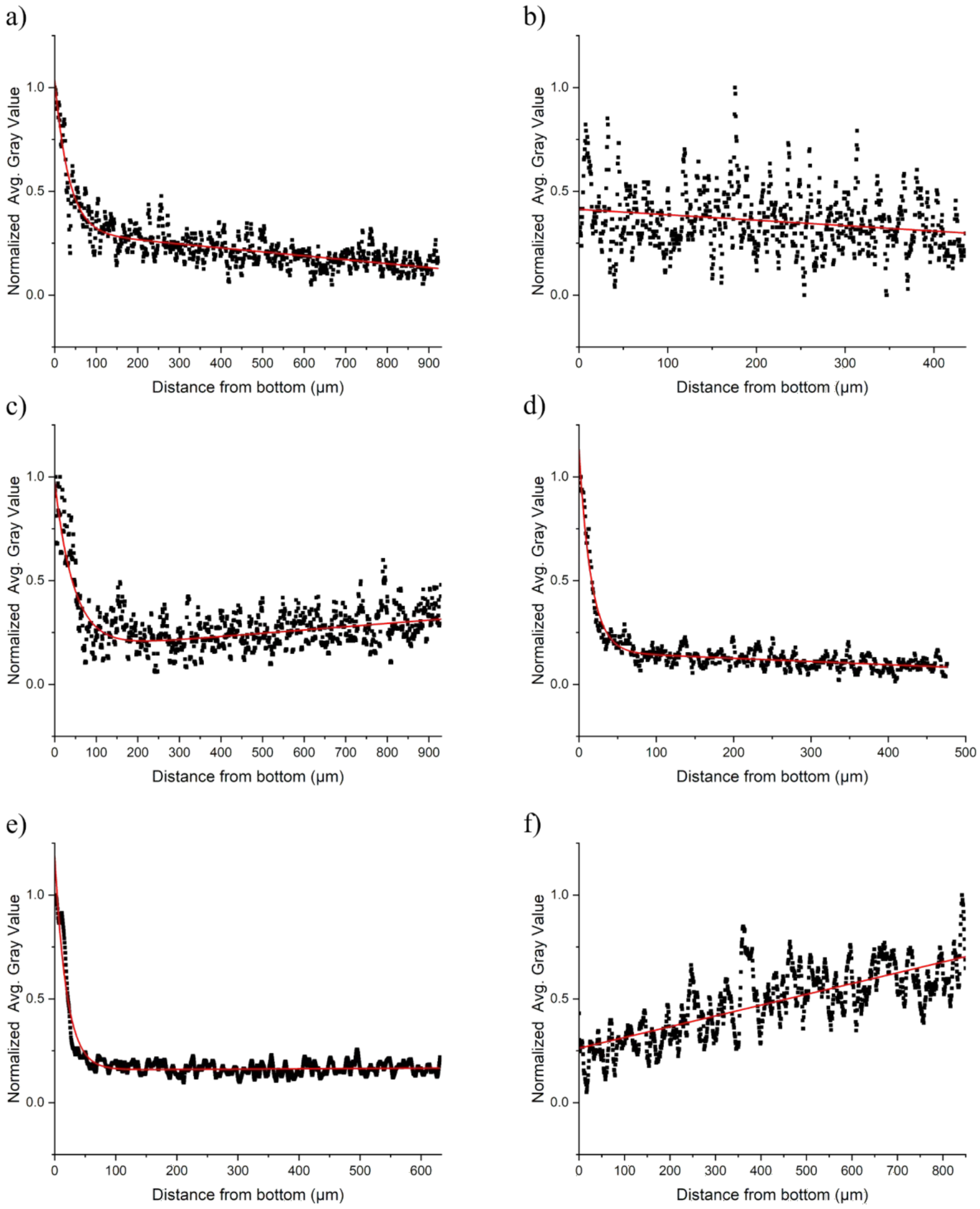

Figure S8. Normalized average gray values from EDS images vs distance from the bottom of films for (a) HDI 30_{25RH}, (b) HDI 40_{25RH}, (c) IPDI $30_{1.1TEA}$, (d) IPDI $50_{1.1TEA}$, (e) IPDI $50_{0.75TEA}$, and (f) IPDI $50_{0.25TEA}$. Data are fitted to either exponential linear combination or linear equation.

Table S7. Line functions used to fit gray values including the different rates of gray value changes and percentages of change in total gray value (GV) due to each part of the line (exponential and linear portions).

Sample	Equation Fit	% Exp	Exp Rate (GV/µm)	% Linear	Linear Rate (GV/µm)
HDI 30	Exponential Linear	73%	-2.93E-02	27%	-1.89E-04
HDI 40	Linear	-	-	100%	-2.61E-04
IPDI 30	Exponential Linear	81%	-2.17E-02	19%	1.58E-04
IPDI 50	Exponential Linear	98%	-6.39E-02	2%	-1.54E-04
IPDI $50_{0.75}$	Exponential Linear	100%	-5.23E-02	-	-
IPDI $50_{0.25}$	Linear	-	-	100%	5.19E-04

Exponential Linear: $y = p_1 e^{-\frac{x}{p_2}} + p_3 + p_4 x$
p_1 = coefficient, p_2 = unknown, p_3 = offset, and p_4 = coefficient
Linear: $y = \beta_0 + \beta_1 x$
β_0 =intercept and β_1 =slope.

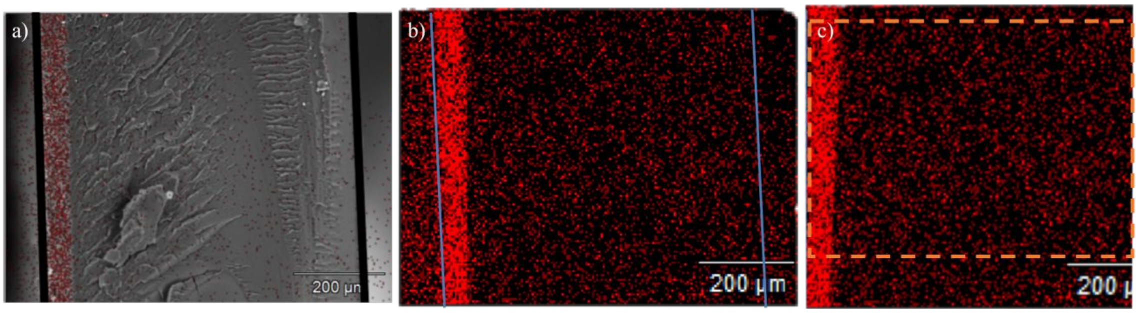

Figure S9. Example of steps of data analysis process utilizing ImageJ for IPDI $50_{0.75}$ composite. (a) Raw SEM image with edges marked, (b) raw EDX Fe map of the same scale with edges marked, and (c) EDX Fe map image with edges cropped and image skewed. Dash orange lines indicate the areas where gray values were measured.

Raw SEM images (Figure S9a) were aligned with Fe EDX maps that excluded any surface features (Figure 9b). Edges were marked on EDX images based on their location seen in raw SEM images. This image was then cropped to remove sections of the image of Fe signals not found on the surface of the cross-section. The image was then skewed to align both the bottom (left side of image) and top of the films (right side of image) to be parallel. All image transformations were done through GIMP image processing software. These images were then loaded into ImageJ and the scale was set by placing a line on the scale bar within the saved image and clicking the "Set Scale" function found within the "Analyze" tab. Images were then converted into 16-bit format and the threshold was set as Fe signals will be displayed as black and the background as white. Sections were drawn as rectangles to include most of the film without capturing elements in the figure such as the scale bar as seen in Figure S6c. After the selection was made, a profile plot was made by utilizing the function of the same name in the "Analyze" tab. This would return data of the average gray values of the full width of the cross-section (column from the top and bottom of the image) at each pixel length. These lengths were converted µm units based on the set scale. This data was exported into a tabulated format and processed with OriginPro data processing software. The average gray values are proposed to be representative of the concentration of FRGO.
